# Tackling the Antimicrobial Resistance “Pandemic” with Machine Learning Tools: A Summary of Available Evidence

**DOI:** 10.3390/microorganisms12050842

**Published:** 2024-04-23

**Authors:** Doris Rusic, Marko Kumric, Ana Seselja Perisin, Dario Leskur, Josipa Bukic, Darko Modun, Marino Vilovic, Josip Vrdoljak, Dinko Martinovic, Marko Grahovac, Josko Bozic

**Affiliations:** 1 Department of Pharmacy, University of Split School of Medicine, Soltanska 2A, 21000 Split, Croatia; drusic@mefst.hr (D.R.); aperisin@mefst.hr (A.S.P.); dleskur@mefst.hr (D.L.); jbukic@mefst.hr (J.B.); dmodun@mefst.hr (D.M.); 2 Department of Pathophysiology, University of Split School of Medicine, Soltanska 2A, 21000 Split, Croatia; marko.kumric@mefst.hr (M.K.); marino.vilovic@mefst.hr (M.V.); josip.vrdoljak@mefst.hr (J.V.); dinko.martinovic@mefst.hr (D.M.); 3 Laboratory for Cardiometabolic Research, University of Split School of Medicine, Soltanska 2A, 21000 Split, Croatia; 4 Department of Maxillofacial Surgery, University Hospital of Split, Spinciceva 1, 21000 Split, Croatia; 5 Department of Pharmacology, University of Split School of Medicine, Soltanska 2A, 21000 Split, Croatia; marko.grahovac@mefst.hr

**Keywords:** artificial intelligence, machine learning, antimicrobial resistance, drug discovery

## Abstract

Antimicrobial resistance is recognised as one of the top threats healthcare is bound to face in the future. There have been various attempts to preserve the efficacy of existing antimicrobials, develop new and efficient antimicrobials, manage infections with multi-drug resistant strains, and improve patient outcomes, resulting in a growing mass of routinely available data, including electronic health records and microbiological information that can be employed to develop individualised antimicrobial stewardship. Machine learning methods have been developed to predict antimicrobial resistance from whole-genome sequencing data, forecast medication susceptibility, recognise epidemic patterns for surveillance purposes, or propose new antibacterial treatments and accelerate scientific discovery. Unfortunately, there is an evident gap between the number of machine learning applications in science and the effective implementation of these systems. This narrative review highlights some of the outstanding opportunities that machine learning offers when applied in research related to antimicrobial resistance. In the future, machine learning tools may prove to be superbugs’ kryptonite. This review aims to provide an overview of available publications to aid researchers that are looking to expand their work with new approaches and to acquaint them with the current application of machine learning techniques in this field.

## 1. Introduction

Antimicrobial resistance is recognised as one of the top threats healthcare is bound to face in the future. There have been various attempts to preserve the efficacy of existing antimicrobials, develop new and efficient antimicrobials, manage infections with multi-drug resistant strains, and improve patient outcomes, resulting in a growing mass of routinely available data including electronic health records and microbiological information that can be employed to develop individualised antimicrobial stewardship [1].

As science has evolved, there has been an overlap between fields, and researchers are seeing more benefits from utilising recent advancements in computational methods, machine learning, and artificial intelligence (AI) development. These technologies are rapidly being incorporated across various disciplines and almost every research area to accelerate scientific discoveries. As they allow the optimal utilisation of large amounts of data, for example, clinical and laboratory data, and can support evidence-based decision making, they substantially save time on research [1,2,3,4].

High-quality machine learning models rely on comprehensive, structured, well-curated datasets [5]. When adequately designed and trained, models can extract meaningful rules and develop predictive tools; they learn objectively and, more often than not, make more accurate predictions compared with those seen in everyday practice [1]. Ultimately, the goal is to develop a system that autonomously improves its performance and accuracy without programming [6]. Such systems would substantially contribute to easing the burden of antimicrobial resistance [7].

A recent study revealed a substantial increase in publications and research on the use of machine learning in the field of antimicrobial resistance [8]. These methods have broad applications in microbiology, from diagnostics, drug and vaccine discovery and applications in epidemiology [1,3,9].

In biomedical research, in the past ten years, broad application of artificial intelligence, deep learning, and machine learning methods can be seen across the field. This narrative review highlights some of the remarkable opportunities that machine learning offers when applied in research related to antimicrobial resistance, including genome analysis for prediction of resistant strains, susceptibility testing, drug discovery, and potential clinical applications. Due to the exponential number of publications on the topic of machine learning applications in the field of antimicrobial resistance, this review aims to provide an overview rather than an in-depth analysis of available publications to aid researchers that are looking to expand their work with new approaches and to acquaint them with the current application of machine learning techniques in this field.

## 2. Genome Analysis for Prediction of Resistant Strains and Susceptibility Testing

Whole-genome sequence data and susceptibility profiles of different microorganisms can be used to train models to predict resistance in bacteria [10,11,12]. Machine learning has been widely utilised in predicting antimicrobial resistance genes and translating genetic mechanisms [13,14,15,16,17,18,19,20,21]. Machine learning based on protein sequences successfully predicted antimicrobial resistance genes in Gram-negative bacteria with over 90% accuracy [22,23]. Researchers investigating biomarkers for *Pseudomonas aeruginosa*’s carbapenem resistance implemented a learning model using a gradient boosting decision tree (GBDT) algorithm to screen for important glycan structures associated with resistant strains [24]. A machine learning model was successfully implemented in research for the classification of resistant genomes in *Pseudomonas aeruginosa*. According to the authors, the machine learning classifier had more predictive power for resistance to individual carbapenems than the broader mechanistic categories, as it included even the uncharacterised mutations found on the genes associated with these mechanisms. Furthermore, it identified several specific features relevant to predicting resistance, although these were not present in greater frequency among the resistant isolates [25]. Resistance prediction models of *Pseudomonas aeruginosa* for imipenem and meropenem were constructed using machine learning techniques paired with next-generation sequencing through matching resistance genotypes with phenotypes. Such models can be used to directly predict carbapenem resistance in clinical samples [26]. Machine learning was utilised to determine whether *Elizabethkingia bruuniana* and *Elizabethkingia meningoseptica* strains were resistant or susceptible to different antimicrobials [27]. Using 25 whole-genome sequences, researchers developed a genome-based machine-learning approach for discrimination between vancomycin-intermediate and vancomycin-susceptible *Staphylococcus aureus* [28]. Furthermore, PRAP, the Pan-Resistome Analysis Pipeline, has successfully analysed the genome and identified antimicrobial resistance genes of *Salmonella enterica* isolates [29].

Machine learning can improve the prediction of the antimicrobial resistance phenotype from genomic data [30]. Efforts are being made to make the models more generalisable as training a distinct model for each antimicrobial and species for predicting resistance may miss some valuable opportunities [31]. When integrating machine learning into research, antimicrobial resistance genes are often identified that have never previously been associated with resistance in any microbe [32,33]. Machine learning techniques can analyse large datasets much faster than traditional statistical methods. This is particularly relevant in genomics, where whole genome sequencing data is used to predict antibiotic resistance. Machine learning can swiftly identify patterns or resistance genes that would take humans much longer to find. Moreover, machine learning models, once trained, can predict antibiotic resistance from genomic data almost instantly. This is much faster than culture-based methods, which can take days [30,31,32,33].

Moreover, successfully developed novel machine learning models for predicting resistance of *Escherichia coli* to colistin have identified some previously unknown colistin-resistant biomarkers [34]. Supervised machine learning classifiers identified known and unknown resistance-associated mutations and genes related to resistance to 28 antimicrobials in *Escherichia coli* and *Salmonella enterica* [35]. Machine learning can be utilised to successfully identify known and potential new antimicrobial genes in *Neisseria gonorrhoeae* [36]. DeepARG is an example of a model developed for predicting antimicrobial resistance based on metagenomic data [37] (Table 1). These techniques have allowed the prediction of antimicrobial resistance based on genome sequences [38,39].

A reservoir of resistance genes may come from previously unknown sources. Other than identifying known resistance genes, machine learning algorithms may be utilised to identify novel yet unrecognised resistance genes [40]. Artificial intelligence has the potential to predict the presence and spread of antimicrobial resistance genes within and across populations [4]. Machine learning was successfully implemented for the identification of antimicrobial resistance genes retrieved from strains on the International Space Station’s environmental surfaces, overcoming traditional cut-offs based on high similarity in DNA sequencing and expanding the catalogue of antimicrobial resistance genes, creating opportunity for alternatives to the current gather-and-return sampling model used for the International Space Station [41].

Many machine learning systems have been developed for the field of clinical microbiology, a number of them for antimicrobial susceptibility testing [4,42]. There is an opportunity for utilisation of machine learning methods in the design and development of sequence-based diagnostics that also predict antimicrobial susceptibility, hence enabling tailored treatment [4,43]. These methods have been utilised to develop tools that predict resistance to a specific antimicrobial, i.e., rifampicin [44] (Table 1). A multi-component, microscopy-based approach that included a deep learning method for automated image classification and prediction of antimicrobial minimal inhibitory concentrations was proposed [45]. Minimum inhibitory concentrations of ciprofloxacin against *Escherichia coli* were successfully predicted based on different mutations [46] (Table 1). There has been much research on machine learning for the prediction of minimum inhibitory concentrations and the determination of resistant strains of different pathogens [47] (Table 1). This can be further developed to predict multi-drug resistance in pathogens [48] (Table 1). Machine learning techniques were adopted to support research into bacterial resistance to a panel of antimicrobials using whole-genome sequence data of *Pseudomonas aeruginosa*, with more than 95% accuracy [49] (Table 1). Furthermore, a similar system was used for the identification of methicillin resistance of *Staphylococcus aureus*, with an accuracy of 87.6%, sensitivity of 91.8%, and specificity of 83.3% [50] (Table 1). In other research, machine learning models were able to predict the minimum inhibitory concentrations of ten different antimicrobial agents for *Staphylococcus aureus* [51] (Table 1). Machine learning models have been utilised to predict minimum inhibitory concentrations of cefixime, ciprofloxacin, and azithromycin against *Neisseria gonorrhoeae* [52].

Whole-genome sequencing data have been successfully used to train models to predict minimum inhibitory concentrations of different antimicrobials against different pathogens, for example, *Klebsiella pneumoniae* [53]. Machine learning based on whole-genome sequencing was used to predict the resistance of four antimicrobials, ciprofloxacin, ceftazidime, cefotaxime, and gentamicin, to *Escherichia coli* [54,55]. 

Integrating traditional machine learning with deep learning models was used to predict minimum inhibitory concentrations of 15 antimicrobials against *Salmonella* [56]. Another study predicted minimum inhibitory concentrations of 15 antimicrobials for nontyphoidal *Salmonella* [39]. With the application of a machine learning feature-selection approach on a *Salmonella enterica* pan-genome, researchers could predict minimum inhibitory concentration values with very high accuracy [57]. Researchers utilised three machine learning algorithms to estimate minimum inhibitory concentrations of 13 antimicrobials against *Acinetobacter baumannii* [58] (Table 1). Furthermore, using multi-branch CNN and Attention model, a deep learning method outperformed traditional machine learning methods when applied in the prediction of the minimum inhibitory concentrations of peptides with antimicrobial activity against *Escherichia coli* [59] (Table 1). However, when several different machine learning models were designed for the prediction of resistance or susceptibility to ciprofloxacin, ceftazidime, and meropenem, some achieved lower accuracy depending on the input data used for training [60] (Table 1). Several available datasets have been used in the training of machine learning models [61]. Most antimicrobial resistance-related machines are not yet ready for implementation in real-life settings. Implementing these methods would only be feasible with further improvement in accuracy and a reduced financial cost, especially for pathogens that take longer to culture [62]. Models that accurately predict antimicrobial resistance phenotypes from genotypes are growing and being further optimised [63,64]. With more significant amounts of data, the performance of machine learning models improves [65]. A deep transfer learning model was developed to overcome the known pitfalls of machine learning when operating with limited datasets. This model achieved accurate and robust antimicrobial resistance predictions based on small, imbalanced datasets [66].

Raman spectroscopy was enhanced with machine learning techniques for identification and antimicrobial susceptibility testing [67]. When surface-enhanced Raman spectroscopy was coupled with machine learning methods, it detected virulence and carbapenem resistance in *Klebsiella pneumoniae* [68,69] (Table 1). Furthermore, it was successfully employed to differentiate resistant and susceptible strains of *Escherichia coli* [70]. Rapid identification of methicillin-resistant Staphylococcus aureus is possible when surface-enhanced Raman spectroscopy is coupled with deep learning methods [71].

Coupled with matrix-assisted laser desorption/ionisation and time-of-flight mass spectrometry (MALDI-TOF MS), machine learning has successfully been implemented for antimicrobial susceptibility testing and prediction of antimicrobial resistance in pathogens [72,73]. Examples include identifying antimicrobial resistance to benzylpenicillin or multidrug *Staphylococcus aureus* isolates and development of high-performance classifiers for ciprofloxacin and tetracycline-resistant *Campylobacter jejuni* and *Campylobacter coli* isolates [74,75]. Methods have been developed to distinguish vancomycin-intermediate *Staphylococcus aureus* from vancomycin-susceptible *Staphylococcus aureus* [76,77]. A convolutional neural network model was developed to rapidly predict vancomycin-resistant *Enterococcus faecium* in clinical samples after analysis of the matrix-assisted laser desorption ionisation time-of-flight mass spectrometry peaks’ pattern [78]. MALDI-TOF MS spectra coupled with a machine learning algorithm rapidly predicted the susceptibility of *Enterococcus faecium* to vancomycin with a mean accuracy of 0.78 [79].

Artificial intelligence and machine learning have been implemented to diagnose microorganisms and predict susceptibility to antimicrobial agents [80]. Researchers in China investigating the *Pseudomonas aeruginosa* ST316 sublineage causing ear infections used the K-mer machine learning approach to generate predictive models and identify biomarkers of resistance to gentamicin, fosfomycin, and cefoperazone–sulbactam [81]. A machine learning approach using mutations in *Neisseria gonorrhoeae* was proven helpful in predicting ceftriaxone susceptibility and decreased susceptibility. From seven investigated models, the random forest classified model showed the highest performance [82]. Machine learning was utilised for differentiating *Enterobacter cloacae* complex species [83]. 

Machine learning algorithms were implemented while developing antimicrobial resistance prediction models for *Escherichia coli* [84,85]. These algorithms were successfully utilised to enhance methods for discrimination between piperacillin/tazobactam-resistant and susceptible *Escherichia coli* isolates [86]. Furthermore, deep learning of single-cell subcellular phenotypes was utilised for determining antimicrobial susceptibility in *Escherichia coli* for ciprofloxacin, gentamicin, rifampicin, and co-amoxiclav [87].

Machine learning techniques have been successfully applied to distinguish the methicillin-resistant form of methicillin-sensitive *Staphylococcus aureus* [88,89]. A deep residual learning framework was trained for the classification of bacteria samples. The model predicted *Staphylococcus aureus*, *Klebsiella pneumoniae*, and *Bacillus subtilis* classes with 100% sensitivity. Such attempts may enable bacterial identification, avoiding cell culturing [90]. Furthermore, researchers have developed a mNGS-based machine learning model for rapid antimicrobial susceptibility testing of *Acinetobacter baumannii* that may come to take the place of time-consuming culture-based procedures [91]. This may be particularly beneficial for pathogens that require long culturing, such as *Mycobacterium tuberculosis* [92].

Machine learning classifiers can predict resistant and susceptible phenotypes of *Mycobacterium tuberculosis*, *Klebsiella pneumoniae*, and *Salmonella enterica* using bacterial genome sequence data [93]. There are several applications for machine learning in predicting drug resistance of *Mycobacterium tuberculosis* from sequence data [92,94,95,96,97,98,99,100,101]. The deep graph learning method has been used to predict drug resistance in tuberculosis with a sensitivity of over 90% for isoniazid, ethambutol, and pyrazinamide [102]. Incorporating different models into research, potential new candidate mutations in *Mycobacterium tuberculosis* not previously reported in the literature, which can be related to resistance, have been identified [103]. Convolutional neural network applied in the analysis of the *Mycobacterium tuberculosis* genome identified 18 sites not previously associated with antimicrobial resistance [104]. Some tools have been made available to interested researchers. The TuBerculosis Drug Resistance Optimal Prediction tool allows users to input sequencing data for the prediction of resistance in *Mycobacterium tuberculosis* [105]. The Translational Genomics platform for Tuberculosis, GenTB, is a publicly available online tool designed to predict drug resistance in *Mycobacterium tuberculosis* from genomic data [106].

These methods can be used to predict pyrazinamide resistance of *Mycobacterium tuberculosis* and to pinpoint genes and mutations responsible for the resistance [107,108]. A pipeline for developing machine learning models of mycobacterium tuberculosis drug resistance prediction was made publicly available [109]. Moreover, a metabolic allele classifier was developed to predict antimicrobial resistance phenotypes of Mycobacterium tuberculosis [110]. Overall, there have been many applications in drug resistance profiling of tuberculosis [111]. In drug resistance profiling of tuberculosis, some models have achieved 99% accuracy [112]. Machine learning classifiers were used to predict resistance mutations related to rifampicin as an alternative rapid method [113].

Machine learning techniques have been proposed for the prediction of antimicrobial resistance of *Klebsiella pneumoniae* [114]. These models coupled with logistic regression have been successfully used for identification of strong resistance predictors in *Klebsiella pneumoniae.* Such models may guide diagnosis and treatment selection in clinical practice [115]. Analysis of genomic data with the implementation of machine learning has allowed predictoin of resistance to polymyxins in *Klebsiella pneumoniae* [116].

Machine learning technologies can be implemented for rapid differentiation of resistant *Mycobacterium abscessus* complex subspecies from macrolide-susceptible subspecies [117]. These technologies have been used with other methods to investigate mechanisms of resistance in *Pneumocystis jirovecii* [118]. Furthermore, machine learning models were utilised to analyse the drug resistance of *Candida auris* [119].

Machine learning algorithms can identify risk and clinical predictors of multidrug-resistant *Enterobacterales* infections in persons infected with HIV [120]. Moreover, different machine learning approaches have been utilised to predict the drug resistance of HIV [121,122,123,124,125,126,127,128,129,130,131,132]. For example, models were implemented to predict HIV-1 protease resistance [133]. A web application, SHIVA, that can determine drug resistance in HIV has been developed [134]. Furthermore, the freely available web service HVR, based on a validated machine learning model, predicts human immunodeficiency virus type 1 drug resistance to nucleoside and non-nucleoside reverse transcriptase and protease inhibitors [135]. Furthermore, research has demonstrated that deep learning networks are able to differentiate viruses in clinical samples and that the addition of deep learning to single-particle fluorescence microscopy can rapidly detect and classify viruses [136]. There have been efforts to predict antiviral drug resistance of the Influenza virus with machine learning [137]. In research incorporating statistical analysis, a machine learning algorithm was used for predictive analysis of virulence genes to explore further the ones potentially related to specific antimicrobial resistance phenotypes [138].

**Table 1:** Summary of selected studies showing the efficacy of machine learning in AMR prediction.

Study	Pathogen	Algorithm	Target	Result
Aytan-Aktug et al. [10]	*Mycobacterium tuberculosis*, *Escherichia coli*, *Salmonella enterica*, *Staphylococcus aureus*	Neural network	Multiple AMR profile prediction	AUC 0.90–0.95 on test dataset
Chowdhury et al. [22]	*Acinetobacter*, *Klebsiella*, *Campylobacter*, *Salmonella*, and *Escherichia*	SVM	AMR gene classification	>90% accuracy
Pesesky et al. [23]	*Gram-negative Bacilli*	Logistic regression	Antibiotic resistance prediction	Agreement of 90.8% to the phenotypic ASTs
Dang et al. [24]	*P. aeruginosa*	GBT	Predicting glycopatterns for carbapenem resistance	AUC 0.95
Liu et al. [25]	*P. aeruginosa*	Neural network	Predicting imipenem and carbapenem resistance	AUC 0.906 and 0.925
Tian et al. [34]	*E. coli*	Lasso regression	Predicting colistin resistance	AUC 0.902 on validation dataset
Shi et al. [36]	*Neisseria gonorrhoeae*	DNP-AAP	Predicting AMR	AUC 0.97–0.99
Arango-Argoty et al. [37]	Antibiotic resistance genes from multiple pathogens	DeepARG	Predicting ARG	Precision (>0.97), recall (>0.90)
Portelli et al. [44]	*M. tuberculosis*	Linear classifiers, decision trees, ensemble classifiers	Predicting rifampicin resistance	Sensitivity 92.2%, specificity 83.6%
Pataki et al. [46]	*E. coli*	Random forest	Predicting ciprofloxacin minimum inhibitory concentration	AUC 0.99
Valizdeh et al. [47]	*C. jejuni*, *S. enterica*, *N. gonorrhoeae* and *K. pneumoniae*	XgBoost	Predicting AMR	Accuracy 0.95–0.97
Ren et al. [48]	*E. coli*	Random forest	Multi-drug resistance prediction	F score 0.93 ± 0.04
Noman et al. [49]	*P. aeruginosa*	Random forest	Predicting AMR	Accuracy > 0.97
Jeon et al. [50]	*S. aureus*	AMRQuest	Presumptive identification of MRSA	Sensitivity of 91.8%, Specificity of 83.3%, Accuracy of 87.6%
Wang et al. [51]	*S. aureus*	XgBoost, random forest, SVM	Identification of MRSA	Category agreement > 85% and >90% (one-two fold dilution)
Ayoola et al. [56]	*Salmonella* spp.	Genome feature extractor pipeline (combining random forest and MLP)	Predicting MIC	Accuracy > 96%
Gao et al. [58]	*A. baumani*	Random forest, SVM, and XgBoost	Predicting MIC	Average essential agreement 90.90% (95% CI, 89.03–92.77%)
Yan et al. [59]	*E. coli*	MBC-Attention	Predicting AMR	PCC of 0.775 and a root mean squared error (RMSE) of 0.533 (log μM)
Yasir et al. [60]	*P. aeruginosa*	Random forest, and nine other classifiers	Predicting AMR	Accuracy 0.73
Ren et al. [66]	*E. coli*	CNN with transfer learning	Predicting AMR	Validation AUC 0.72–0.93
Lu et al. [68]	*K. pneumoniae*	CNN on Raman spectroscopy	Predicting AMR	AUC 0.97

SVM—support vector machine, AMR—antimicrobial resistance, ASTs—antibiotic susceptibility tests, AUC—area under the ROC curve, DNP-AAP—(deep neural pursuit—average activation potential), DeepARG—deep learning antibiotic resistance gene, XgBoost—Extreme Gradient Boosting machine, MLP—multi-layered perceptron (neural network), MIC—minimal inhibitory concentration, MBC-Attention—a combination of multi-layered convolutional networks and attention mechanism, PCC—Pearson correlation coefficient, RMSE—root mean squared error, CNN—convolutional neural network.

## 3. Drug Discovery

**Figure 1:**
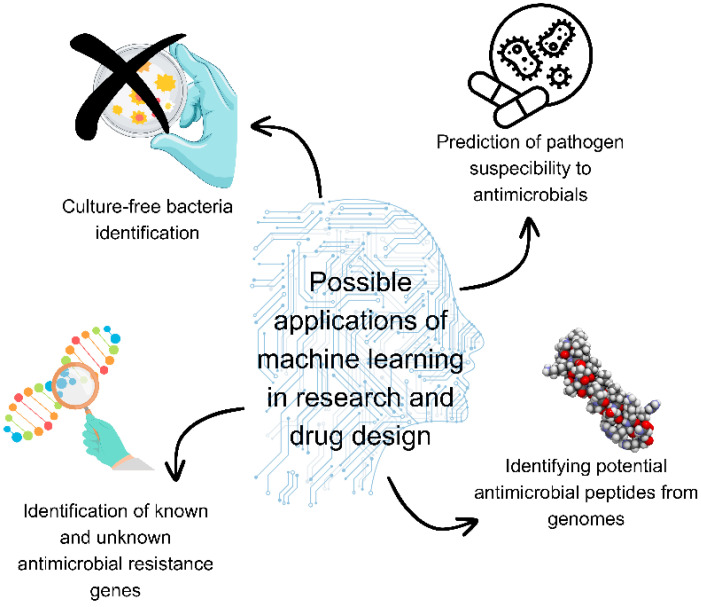
Possible applications of machine learning in research and drug design. Figure was made using Canva.

Machine learning may find great application in rapid antimicrobial resistance predictions, and it may enhance the discovery of novel antimicrobials [66] (Figure 1). In research on antimicrobial peptides, support vector machines, random forest machine learning algorithms, deep neural networks, and convolutional neural networks have found application [139]. Previously developed machine learning models were used to screen a chemo library and identify potential drug candidates for known therapeutic targets [140]. Traditional machine learning may be used to discover antimicrobial peptides in large-scale natural known peptide libraries, while artificial neural networks may predict peptide activity against pathogens and identify highly active peptides. Furthermore, models may be used for design by optimisation and in de novo design of antimicrobial peptides [141]. Machine learning models may be used as aids in screening during the early stages of designing peptide-based antimicrobials [142,143,144,145,146,147,148,149,150]. Machine learning algorithms allow rapid in silico screening and classification of antimicrobial peptides for investigating activity against specific species, providing savings and replacing or enhancing time-consuming conventional methods [151]. These techniques can be used to screen libraries of compounds to identify drug candidates. This approach was used to discover beta-lactamase CMY-10 inhibitors against *Enterobacteriaceae* [152].

Machine learning techniques have been widely applied in research to develop and enhance antimicrobials [153]. A machine learning model, CalcAMP, can predict the antimicrobial activity of peptides against both Gram-positive and Gram-negative bacteria [154]. An antimicrobial peptide classification model, AMP-BERT, was developed as a tool for pre-screening for antimicrobial drug candidates that may promote the discovery process of antimicrobial drugs [155]. Deep-AmPEP30 is a prediction tool to identify short-length antimicrobial peptides from genomic sequences [156]. However, antimicrobial peptides can be identified from various organisms [157]. Machine learning methods were implemented as tools to identify possible embedded antimicrobial peptides from different proteomes [158]. Deep learning models can be implemented for predicting antimicrobial peptides in peptide sequences, such as AMPlify, which identified four antimicrobial peptides in the bullfrog genome [159,160]. 

To overcome the fact that computational approaches are usually unable to predict the functional activities of antimicrobial peptides, researchers recently developed a deep learning approach, iAMPCN, that improved the prediction of antimicrobial peptides’ functional activities [161]. A proposed model of NIRBMMDA was developed for the prediction of potential microbe–drug association and has shown promising results. For example, 17 of the top 20 predicted microbes for ciprofloxacin were identified and confirmed in published literature. However, such models’ performance depends on the dataset used to train the model [162]. Furthermore, machine learning was utilised in research into the structure–activity relationship of New Delhi metallo-beta-lactamase-1 inhibitors, setting a path for further drug discovery and drug optimisation [163].

Machine learning techniques can be incorporated into all stages of drug discovery, including predicting biological responses. Machine learning has been utilised to guide decision making during the de novo design and differentiation of molecules with desired properties. Models have been applied to accelerate the discovery and design of drug candidates against different multidrug-resistant microbes. They may significantly cut the conventional cost and screening efforts required for the process of screening for new drug candidates [164].

Several tools have been developed for identification of lead compounds against *Mycobacterium tuberculosis*, for prediction of the potential toxicity of a compound of interest, and for classifying drug resistance in isolates and linking specific mutations with resistance [165]. Machine learning techniques have been utilised to predict the antitubercular effects of drugs [166]. Researchers have developed a computational model (termed iAMAP-SCM) that can identify and characterise peptides with antimalarial activity using just sequence information [167]. Furthermore, machine learning techniques have been utilised to design novel peptide sequences with expected antibacterial activity against *Escherichia coli* [168]. A similar method was employed to identify an antimicrobial peptide active against *Staphylococcus epidermidis* [169].

Available machine-learning algorithms have been used for assessing the theoretical function of newly designed antimicrobial peptides [170]. In research, deep learning and computational methods were utilised to create and prioritise de novo antimicrobial peptides, which showed no development of resistance in vitro [171]. Moreover, chemically modified peptides can be analysed to predict their potential antimicrobial activity [172].

Computational tools have been used for possible target identification, namely, quorum-sensing peptides [173]. Machine learning algorithms may be utilised to predict molecular properties of novel efflux pump inhibitors, as it is now essential to screen for therapeutic targets capable of restoring the effectiveness of known antimicrobials [174]. Furthermore, models were used in quantitative structure–activity relationship (QSAR) modelling of LpxC inhibitors to predict the inhibitory activity and identify the best model [175].

An interpretable machine learning algorithm, InterPred, can associate a bioactive molecule with its bioactive moiety that has a new mechanism of action and can further guide the selection of candidate molecules in drug repurposing and enable prioritisation of entities with novel mechanisms of action [176]. Moreover, the development of the novel antimicrobial halicin that has demonstrated in vitro and in vivo efficacy against the high-priority pathogen *Acinetobacter baumannii* was supported with machine learning models [4]. This drug was repurposed, as originally it was being developed as an anti-diabetic agent; however, an AI-supported analysis showed it has a bactericidal effect after screening via the Drug Repurposing Hub [2].

PmxPred was utilised as a predictive tool and may be useful in developing next-generation polymyxins as it accelerated identification of polymyxin analogues active against Gram-negative bacteria [177].

Machine learning models were utilised to predict peptides with biofilm inhibition activity [178]. Such models have been developed to mine peptide databases, classify peptides that may have potential antibiofilm activities, and identify the characteristics of current antibiofilm peptides. For example, the Extreme Gradient Boosting model has predicted peptides with an over 98% accuracy [179]. A machine learning multi-technique consensus workflow was developed to predict the protein targets in molecules with confirmed inhibitory activity against biofilm formation by *Pseudomonas aeruginosa* [180].

Machine learning techniques can also be used to predict the toxicity of antimicrobial peptides, which can present a notable challenge in their clinical implementation [181,182]. Today, there is a freely available microbial strain-specific antimicrobial peptide predictor that is based on published research and can make predictions of general antimicrobial, antibacterial, antifungal, antiviral, and haemolytic activity of peptides [183].

Machine learning techniques have been utilised to discover bacteriocins [184]. The peptides bacteriocins, produced by bacteria in their metabolic process during ribosome synthesis, are an appealing substitute for conventional antimicrobials, as they are highly specific. Researchers presented a bacteriocin prediction pipeline based on machine learning and developed an application called BPAGS so that users can test different protein sequences for bacteriocin prediction without prior programming knowledge [185]. BaPreS achieves a prediction accuracy of 95.54% for testing protein sequences. It was developed as a prediction tool to discover new highly dissimilar bacteriocins that may be further developed into highly effective antimicrobial drugs [186].

Machine learning techniques allow the in silico screening of compounds that may yield antimicrobial effects and then guide further in vivo tests [187]. Supervised learning techniques (like random forest and neural networks) were employed to identify bacterial cell wall lyases as candidates with antibacterial properties [188]. The models were trained using large datasets comprising chemical properties and biological activity data of known antibacterial agents, enhancing their ability to identify promising candidates effectively [188].

DefPred has been developed for the classification and identification of defensins, a group of antimicrobial peptides [189].

Machine learning methods have enabled fast identification of potential bacteriophages [190], and have been implemented to predict phage virion proteins with 83% accuracy, 82% sensitivity, and 89% specificity [191]. Furthermore, a machine learning tool was used for the targeted identification of phage depolymerase, which is likely to be a powerful weapon against antimicrobial-resistant bacteria [192].

Machine learning tools for the design of antimicrobial peptides are being further optimised to achieve higher precision and selectivity for resistant targets [193]. They are an essential tool for scientists to identify critical properties of structures [177]. AI accelerates the timeline of research through efficient analysis of enormous datasets and supports innovating strategies that reduce costs and accelerate the development of new drugs. The introduction of AI may necessitate only 2 years for the development of new antimicrobials. New antimicrobials can outperform existing compounds as they can evade resistance, possess anti-biofilm activities, and have new modes of action. Furthermore, AI can make estimations on further efficacy, resistance trends, and adverse reactions, thus prioritising the most promising compounds [164,194].

## 4. Potential Clinical Applications

Inappropriate treatment inevitably leads to unfavourable outcomes and is rooted in an inability to rapidly identify patients who are at risk of infection with the antimicrobial-resistant pathogen [1]. Machine learning models based on electronic health records have been used to predict bacterial infections and optimal antimicrobial treatment in hospitalised patients [195]. These algorithms have seen application in predicting risks of developing multi-drug resistant infection with Gram-negative bacteria to guide treatment choice and predict patients’ outcomes [196]. However, model training is crucial. There have been various attempts to implement models in clinical settings. One example was predicting perirectal colonisation with carbapenem-resistant *Enterobacteriaceae* (CRE) and other carbapenem-resistant organisms at hospital unit admission. However, the developed decision tree models did not achieve satisfactory results [197].

As machine learning is mathematically oriented, it does not depend on prior knowledge of resistant strains and has proven valuable in predicting antimicrobial resistance. As such, it may assist in informed drug decisions [198]. Adequately trained artificial intelligence successfully predicted the probability of antimicrobial resistance in patients, taking patients’ characteristics, admission data, historical drug treatments, and culture test results into account [199]. Another model was developed for the estimation of patients’ length of stay, mortality, and outcomes on any given day should the antimicrobial treatment be continued or stopped. In the mentioned study, results suggested that stopping antimicrobials earlier may be associated with shortening patients’ hospitalisation, indicating savings for healthcare. Such a model may be used as a proxy for treatment optimisation [200]. Algorithms were developed for the optimal dose of meropenem and polymyxin B against carbapenem-resistant *Acinetobacter baumannii* based on in vitro data [201].

A review from 2020 covering this topic identified 60 different machine learning clinical decision support systems developed for diagnosis or prediction of infection, prediction of treatment response or antimicrobial resistance, choice of antimicrobial or antiretroviral therapy, or early detection or stratification of sepsis. Some questions arose in the research regarding ideal machine learning systems for aiding clinical decision in reducing risk of antimicrobial resistance. The researchers concluded that none of the systems included data on local antimicrobial resistance rate. Moreover, they used somewhat fewer data than a clinician would [202]. Systems to aid diagnosis and distinguish bacterial from viral infection can greatly reduce unnecessary antimicrobials in dominantly viral infections. However, due to the imperfections of these systems, different patient variables that may be taken into consideration, different learning techniques, etc., they may currently be considered as support for clinical decision rather than an alternative. The development of such aids for clinical practice requires open access to databases for training these systems. Furthermore, great implementation in the future may be expected only should they be made financially accessible [202]. 

Machine learning models developed to predict antimicrobial susceptibility based on electronic health data have demonstrated they can optimise antimicrobial treatment and reduce the use of second-line antimicrobials [203]. The literature reports that machine learning-based algorithms in clinical settings can help reduce prescription of unnecessary antimicrobials by up to 40% [204]. Furthermore, machine learning methods successfully predicted whether patients were infected with extended-spectrum β-Lactamase–producing bacteria, with over 90% positive and negative predictive value [205]. Researchers have utilised machine learning-based clinical decision support systems in efforts to standardise decisions on when to switch patients from IV to oral antimicrobials [206]. As machine learning and artificial neural network models have been utilised for the detection of carbapenem-resistant *Klebsiella pneumoniae*, they may serve as a screening tool in clinical practice for rapid identification [207]. Moreover, approaches have been made combining biochemical markers and microbiology susceptibility tests to predict infection risk. Such analyses can easily be incorporated into everyday clinical work [208].

Different algorithms assessing antimicrobial susceptibility have been tested to improve empirical treatments in intensive care unit centres and avoid time-consuming conventional analyses [209,210]. Other than susceptibility testing, several other clinical applications of machine learning algorithms are described in the literature. Machine-learning algorithms applied directly to clinical samples may be utilised to accurately define effective antimicrobial therapy [211]. In antimicrobial stewardship, machine learning was employed for the identification of risk factors associated with the development of ventilated hospital-acquired pneumonia and mortality. Such models may enable early detection of at-risk patients and guide treatment decisions [212]. Moreover, machine learning models have been developed to help clinicians determine when an antimicrobial is unnecessary in the treatment of uncomplicated upper respiratory tract infections in the emergency department [213].

Machine learning models based on electronic health records can be used to aid clinicians in predicting antimicrobial-resistant urinary tract infections [214]. Attempts have been made to predict antimicrobial resistance based on the personal clinical history of patients [215]. Dsaas is a machine learning model developed to help clinicians identify patients at risk of contracting a multidrug-resistant urinary tract infection [216]. Furthermore, attempts have been made to identify biomarkers relevant for accurate point-of-care diagnosis of urinary tract infections, using machine learning models to examine large quantities of data [217]. Moreover, point-of-care testing of antimicrobial susceptibility of *Escherichia coli* in urine coupled with machine learning algorithms dramatically shortens the time needed for analysis [218]. Machine learning analysis of urinary tract and wound infections could predict treatment-induced resistance. Such algorithms can make personalised treatment recommendations [219]. Algorithms have been developed to predict antimicrobial resistance in uropathogens, lowering the probability of using ineffective antimicrobials in the emergency department by 20%. As such, they may aid in empirical treatment choices [220].

Research has demonstrated that AI models can promote the early prediction of urine tract infections and secondary bloodstream infections. In practice, such models can reduce the risk of delayed introduction of antimicrobial treatment in patients with nonspecific symptoms [221]. A supervised machine learning algorithm was adopted to diagnose bacterial infection based on routine blood parameters upon presentation to the hospital [222]. Machine learning to predict antimicrobial resistance in *Escherichia coli* has proven more efficient than laboratory testing. Researchers have developed a machine learning-based online tool that aids clinicians in predicting the resistance phenotype of *Escherichia coli* and accelerates clinical decision-making [223]. In a study by Luterbach et al., a machine-learning model was used to rank the variables predicting 30-day mortality in patients with carbapenem-resistant *Klebsiella pneumoniae* bloodstream infections [224]. Researchers used machine learning to determine changes in risk factors for the prognosis of patients during different periods of the bloodstream infection timeline [225]. Other models may aid in distinguishing commensal strains from *Escherichia coli*, causing bloodstream infections and identifying genetic factors linked to pathogenicity [226]. Furthermore, a machine learning pipeline was established for blood culture outcome prediction that, upon clinical implementation, may reduce the number of unnecessary blood culture tests [227]. 

Machine learning algorithms predicted intensive care unit patients likely to be colonised with resistant pathogens, with sensitivity values above 75% and specificity values ranging from 59% to 83% for different pathogens [228]. Other models were trained to predict resistance to fluoroquinolones in patients with rifampicin-resistant tuberculosis. This may guide treatment selection for these patients. Such models need excellent specificity to avoid unnecessary treatment interventions and additional resistance to alternative treatments. The final model used in this research included information on the prevalence of resistance to fluoroquinolones in the region [229].

Machine learning classifiers were utilised for rapid triage for COVID-19 [230]. A similar approach was employed to differentiate biomarker combinations related to COVID-19 pneumonia [231]. Furthermore, during the COVID-19 pandemic, researchers developed machine learning algorithms that supported the diagnosis of secondary bacterial infection in hospitalised patients. Such interventions may help halt the unnecessary expenditure of antimicrobials in COVID-19 and similar indications [232].

Analysis of hospital data via implementing machine learning techniques and developing clinical decision support tools that predict antimicrobial resistance can serve as a proxy to clinicians in choosing the appropriate antimicrobial, with the ultimate aim of reducing the unnecessary use of antimicrobials [233]. Applying machine learning algorithms to patient data can help guide targeted empirical antimicrobial prescribing [234]. An AI-based, offline smartphone application has even been developed that enables disk-diffusion antibiogram analysis [235]. Integrating artificial intelligence into clinical practice can undoubtedly shorten the time of analysis. For example, deep learning methods have been utilised for the rapid detection of carbapenemase-producing Gram-negative bacteria in only 15 min as an enhanced version of the blue carba test [236]. Although the first applications of machine learning in medicine were seen in the 1980s and 1990s, there is an evident gap between the number of machine learning applications in science and the effective implementation of these systems [237,238]. Most of the proposed solutions aim to enhance, not replace, clinicians’ work, allowing the automatic management of vast amounts of data. However, ideal implementation relies on quality datasets for training as well as training of healthcare personnel to utilise these tools [239].

### Limitations of Machine Learning in Clinical Application

One of the main limitations of machine learning in healthcare and microbiological research is the dependency on high-quality, large-scale datasets. The performance of machine learning models is directly tied to the quality and quantity of the data they are trained on. Issues such as missing data, inconsistent data collection practices, and limited access to diverse datasets can significantly impact model accuracy and generalisability [60]. Many machine learning models, especially deep learning algorithms, are often seen as “black boxes” due to their complexity. This lack of transparency can hinder their acceptance and trust among healthcare practitioners, who may be reluctant to rely on predictions that cannot be easily explained or understood [238].

There is also the issue of model generalisability and the potential for overfitting. Machine learning models can perform exceptionally well on the data they were trained on but may fail to generalise to new, unseen datasets. This is particularly problematic in microbiological research, where the genetic diversity of pathogens and the emergence of new resistance mechanisms can render models quickly outdated. Overfitting to specific datasets can also mislead research directions and clinical decisions. Additionally, during training, models can also learn certain “shortcuts” that lead them to superficially correct classification or prediction [240]. Shortcut learning occurs when a model, rather than comprehending the underlying biological or clinical characteristics that dictate antimicrobial resistance, instead identifies and leverages incidental patterns or anomalies present in the training data to make predictions. These incidental patterns may not be related to the actual biological processes or mechanisms of resistance but can appear statistically significant to the algorithm during the training phase. For example, if a dataset predominantly consists of resistant strains collected from a particular geographic location or a specific laboratory, the model might erroneously associate resistance with location-specific or laboratory-specific metadata rather than the genetic or phenotypic traits that confer resistance. As a result, while the model may perform well on similar datasets, its ability to generalise and accurately predict resistance in strains from different contexts or environments is compromised. This reliance on non-generalisable, dataset-specific quirks rather than true, causative features of resistance can significantly limit the practical application and reliability of machine learning models in predicting antimicrobial resistance across diverse bacterial species and settings [241].

To mitigate shortcut learning, it is crucial to employ diverse and well-curated datasets that accurately reflect the complexity of microbial resistance mechanisms, alongside employing model training strategies that emphasise the learning of biologically relevant features. Additionally, rigorous validation on independent datasets from varied sources is necessary to ensure that the models have genuinely learned to identify the hallmarks of antimicrobial resistance and can make robust predictions across different contexts. Unfortunately, most published research lacks validation [238]. Not all research reports measure performance. Hence, legislation may come in place to set standards for machine learning systems that may be fully implemented in clinical work. As such, they may be regulated as medical devices and may have to include obligatory variables or at least several prespecified patient variables and pass rigorous testing before implementation to reduce possible errors should some clinicians blindly obey the system. Controlled randomised clinical trials evaluating patients’ outcomes are needed to confirm the validity and usefulness of machine learning systems in clinical settings. Furthermore, implementation of such systems may not result in savings for healthcare systems as other staff are needed for maintenance, and they should not be expected to replace clinicians. However, their implementation may result in a decrease in medication errors and other complications arising from suboptimal treatment choices, leading to savings in the long term [202].

## 5. Alternative Applications in the Medical Field

**Figure 2:**
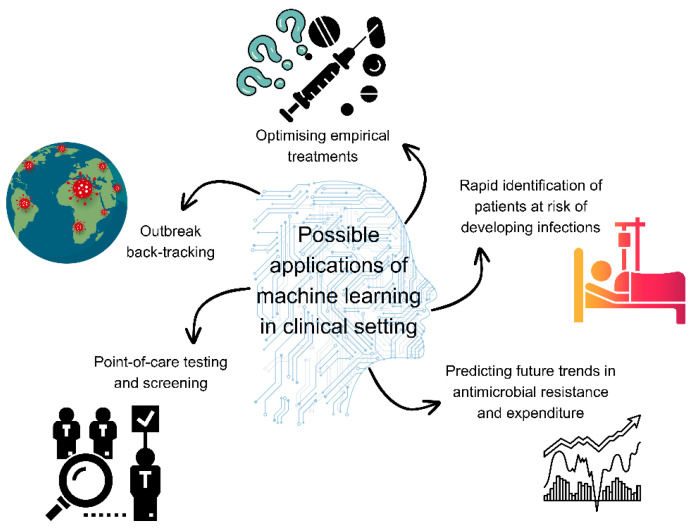
Possible applications of machine learning in the clinical setting. Figure was made using Canva.

AI- or machine learning-enabled devices are seeing wide application in the medical field (Figure 2). Machine learning models can analyse data on antimicrobial resistance and antimicrobial use to identify potential hotspots of resistance and emerging resistance patterns [242]. Models were successfully utilised for mapping antimicrobial resistance threats in marine habitats with more than 75% accuracy [243]. Machine learning may establish itself in antimicrobial stewardship in terms of predicting antimicrobial resistance using genomic and antimicrobial susceptibility testing data (i.e., to identify novel resistance mechanisms and predict resistance from incomplete data) and also as a forecast tool for future changes in resistance rates based on historical prevalence data [6]. Machine learning methods can predict future trends in antimicrobial resistance and expenditure based on current data [244]. Moreover, using machine learning, researchers attempted to predict the future proportions of resistant strains using prior resistance information [245]. Causal machine learning was used to identify critical interventions for the reduction of antimicrobial resistance and outlined that quality of governance and immunisation strategies are vital [246]. In research, machine learning methods have been applied to connect dietary, physiological, and lifestyle features of healthy adults with antimicrobial resistance, suggesting that individuals that had more varied diets, richer in fibre and limited in animal protein, had lower abundances of antimicrobial resistance genes [247].

Machine learning tools can be used in epidemiology to back-track transmission of outbreaks [248,249]. This technique has been applied in identifying the geographical sources of new infections during public health outbreak investigations. A model was trained and implemented to identify and trace *Salmonella enteritidis* infections using whole-genome sequencing data [250]. Furthermore, implementation of machine learning to support logistic regression analysis in outbreak investigation revealed that patients receiving antimicrobials and those older than 55 years were at higher risk for colonisation with vancomycin-resistant enterococci. This was not apparent from the logistic regression [251]. 

Machine learning methods are well established in drug design, and they can also guide the design of pathogen-resistant coatings naturally resistant to biofilm formation [252]. Furthermore, machine learning methods have been utilised to predict the investigation of pathogen attachment to coating polymers for biomedical devices, thus guiding decisions towards the development of devices with less potential risk of complications [253].

## 6. Conclusions

Machine learning methods have been developed to predict antimicrobial resistance from whole-genome sequencing data, forecast medication susceptibility, recognise epidemic patterns for surveillance purposes, propose new antibacterial treatments, and accelerate scientific discovery. Unfortunately, there is an evident gap between the number of machine learning applications in science and the effective implementation of these systems. With more significant amounts of data, the performance of machine learning models improves. High-quality data is a prerequisite for accurate machine-learning based systems; hence, further development requires unrestricted access for all interested researchers.

Possible applications of artificial intelligence-based systems in healthcare are endless. In the way that digitalisation, Google, PubMed, and other search engines have revolutionised libraries and shortened the time needed for accessing and searching data, artificial intelligence can shorten the time needed for complex analyses and much more. In the way that the World Wide Web has facilitated communication and access to information, novel AI-based systems will enable people to speak the language of programming and allow everyone to program according to their specific needs. Machine learning facilitates the optimal use of data for evidence-based decision making. Algorithms can learn objectively and often outperform decisions observed in everyday practice. Machine learning should be able to significantly improve research efficiency, allowing scientists to focus on more complex scientific matters. In the not-so-distant future, training in machine learning may prove to be as essential to researchers as training in statistical analysis, although authors speculate that currently, classical statistical analysis is essential for determining causality [254]. Although the golden era for antimicrobial discovery is far behind us, using AI in the field of antimicrobial resistance may postpone the post-antimicrobial era. Undoubtedly, today is an exciting time to live in. We are looking into a future in which an AI-based system will become highly integrated in biomedical research.

## Data Availability

Not applicable.
